# Cyclic Recurrence of Seizures as a Marker for Super‐Refractory Status Epilepticus

**DOI:** 10.1002/brb3.71396

**Published:** 2026-05-24

**Authors:** Maeva Le Goïc, Mario Chavez, Sophie Demeret, Meriem Bouguerra, Baptiste Crinière‐Boizet, François‐Xavier Lejeune, Lucas di Meglio, Alexandre Ledos, Nicolas Weiss, Vincent Navarro, Virginie Lambrecq

**Affiliations:** ^1^ Paris Brain Institute, INSERM, CNRS, APHP, Pitié‐Salpêtrière Hospital Sorbonne Université Paris France; ^2^ AP‐HP, Sorbonne Université, Hôpital de La Pitié‐Salpêtrière, DMU Neurosciences, Médecine Intensive – Réanimation à Orientation Neurologique Paris France; ^3^ Paris Brain Institute's Data Analysis Core Facility (RRID: SCR_026138), INSERM, CNRS, APHP, Pitié‐Salpêtrière Hospital Sorbonne Université Paris France; ^4^ Université Paris Cité, AP‐HP, Hôpital Lariboisière, DMU Neurosciences, Department of Neurology Paris France; ^5^ Brain Liver Pitié‐Salpêtrière (BLIPS) Study Group, INSERM UMR_S 938, Centre de Recherche Saint‐Antoine, Maladies Métaboliques, Biliaires Et Fibro‐Inflammatoire Du Foie Institute of Cardiometabolism and Nutrition (ICAN) Paris France; ^6^ Groupe de Recherche Clinique En REanimation Et Soins Intensifs Du Patient en Insuffisance Respiratoire aiguE (GRC‐RESPIRE) Sorbonne Université Paris France; ^7^ AP‐HP, Sorbonne Université, Hôpital de La Pitié‐Salpêtrière, DMU Neurosciences, Département de Neurophysiologie Clinique Paris France; ^8^ Sorbonne Université, INSERM, CNRS, Laboratoire d'Imagerie Biomédicale, LIB Paris France

**Keywords:** continuous electroencephalogram monitoring (EEG) monitoring, critical care, cyclic seizures, electroencephalography, status epilepticus

## Abstract

**Background:**

Continuous EEG (cEEG) monitoring during status epilepticus (SE) has enabled the identification of cyclic seizures, characterized by recurrent seizures at regular intervals. However, their clinical implications and prognostic significance remain underexplored. This study aimed to compare patients with cyclic seizures [CSp] and those with noncyclic, sporadic seizures (noncyclic seizures patients [NCSp]) during SE, focusing on clinical features, EEG dynamics, and their impact on survival and functional outcomes.

**Methods:**

In this retrospective study conducted in a tertiary neuro‐ICU, we identified patients who developed cyclic seizures during SE and compared them with patients exhibiting sporadic seizures without a cyclic pattern. All patients underwent continuous‐EEG monitoring for at least 24 h.

**Results:**

Among 138 patients with SE, 12% exhibited CSp, characterized by the absence of clinical correlates, focal onset, and short duration. Seizures recurred every 5–10 min, either early in SE or following controlled epileptic activity. Acute etiologies were more common in CSp than in NCSp (68.8% vs. 6.7%) with autoimmune encephalitis as the predominant cause. CSp patients were younger (31 vs. 52 years) and had no comorbidities but experienced more severe SE. They had super‐refractory SE, required twice as long a stay in the ICU, and had a markedly higher seizure burden (mean of 454 vs. 20 seizures per patient), over the entire monitoring period. The mortality rate reached 30%, and CSp were more likely to develop pharmacoresistant epilepsy, leading to poorer functional outcomes.

**Conclusions:**

Cyclic seizures may represent a critical progression in the evolution of super‐refractory SE, linked to higher seizure burden, increased severity, and worse prognosis. Early detection through cEEG might guide timely therapeutic interventions that could improve patient outcomes in this severe condition.

## Graphical abstract

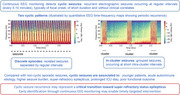

**Abbreviations**
cEEG: continuous video/electroencephalogram monitoring
ASMs: antiseizure medications
CIVADs: continuous intravenous anesthetic drugs
CSA: compressed spectral array
CSp: cyclic seizures patients
GOS: Glasgow Outcome Scale
h: hours
ICU: intensive care unit
IGS II score: index de gravité simplifié II
LOS: length of stay
min: minutes
mRS: modified Rankin Scale
NCSp: noncyclic seizures patients
NORSE: new onset refractory status epilepticus
RSE: refractory status epilepticus
s: seconds
SE: status epilepticusb
SRSE: super refractory status epilepticus

## 1 Background

Status epilepticus (SE) is a severe brain condition characterized by continuous or recurrent epileptic seizures without recovery of consciousness between seizures. It is the second most common neurological emergency after stroke, with high morbidity and mortality, ranging from 10% in responsive cases to 40% in refractory SE (RSE) (Trinka et al. 2015; Rossetti et al. 2024). Prognosis mainly depends on age, comorbidities, underlying etiology, and SE duration (Ferguson et al. 2013; Kirmani et al. 2021; Kämppi et al. 2024). RSE is defined as ongoing seizure activity despite adequate doses of benzodiazepines and at least one additional antiseizure medication (ASM) (Rossetti and Lowenstein 2011). Up to 30% of SE cases become refractory, requiring ICU admission for general anesthesia. Around 15% progress to super‐refractory SE (SRSE), defined as seizure activity that persists or recurs over 24 h after the onset of anesthesia, or that re‐emerges during weaning or attempts to withdraw from sedation (Mayer et al. 2002; Au et al. 2024).

Electrographic non‐motor seizures and electroclinical or electrographic SE associated with coma occur in approximately 20% of patients admitted after SE (Claassen et al. 2004). In this context, continuous video‐electroencephalography monitoring (cEEG) is increasingly used in critical care for early identification, diagnosis, treatment, and management of these patients (Kubota et al. 2018; Limotai et al. 2019; Rubinos et al. 2020; Outin et al. 2021).

The use of cEEG has enabled the identification of a “cyclic” or periodic pattern of seizure recurrence at regular intervals in nearly 20% of SE patients (Friedman et al. 2008; Pinto et al. 2017; Zorlu et al. 2021). Most of these cyclic seizures were electrographic non‐motor seizures (Zorlu et al. 2021). Importantly, these seizures may present as discrete (i.e., single) episodes or as clusters of multiple seizures, raising important questions, as recurrence patterns may reflect distinct pathophysiological mechanisms.

Despite their potential clinical importance, cyclic seizures remain poorly characterized. Further investigation of their electrophysiological and clinical features is essential to improve the understanding and optimize management strategies for SE. No quantitative study has yet examined this clustering pattern, which may be relevant for assessing seizure burden. Furthermore, it remains unclear which patient populations are most affected by cyclic seizures, or whether these seizures occur at specific stages of SE. Patient populations in the literature are heterogeneous, including individuals of all ages, from infants to the elderly, with or without brain injury, admitted for altered mental status, a history of seizures, or SE that did not reach the refractory stage. Approximately 60% of the patients were intubated, with an average ICU stay of 7 days. Given the variability in these factors, along with a limited period of cEEG monitoring, assessing risk factors for cyclic seizures and seizure cluster propensity remains challenging.

Finally, the long‐term prognostic significance of cyclic seizures is poorly understood. Despite conflicting data on the prognostic impact of cyclic seizures, their presence could rather be associated with a poorer outcome (Friedman et al. 2008; Pinto et al. 2017). Patients exhibiting cyclic seizures were more severe and had a worse functional status at discharge and higher mortality during hospital stay when compared with patients without cyclic seizures (Pinto et al. 2017). Nevertheless, in these studies, the control groups either consisted of patients without seizures or patients with continuous and prolonged seizure activity (>30 min) which may limit the ability to isolate the impact of cyclic seizures during SE. Conversely, a more recent study suggested a better outcome in patients under 75 years of age with cyclic seizures compared with noncyclic forms of SE (Zorlu et al. 2021). Whether cyclic seizures impact the long‐term prognosis of SE remains to be addressed.

In our study, the objective was to develop a descriptive and comprehensive framework for understanding this potentially life‐threatening form of SE, characterized by periodic recurrence of ictal events. We characterized the electrophysiological profile of cyclic seizures as well as the clinical profile of patients with cyclic seizures in comparison with those with noncyclic seizures within the context of EEG continuous monitoring. Finally, we assessed the impact of cyclic seizures on survival and functional outcomes by comparing two patient populations, both presenting with seizures.

## 2 Methods

### 2.1 Design of the Study and Population

Adult patients admitted with a clinical suspicion of SE to the neurointensive care unit (ICU) of Pitié‐Salpêtrière University Hospital (Paris), a tertiary care center, between May 2016 and December 2021, who underwent cEEG monitoring for at least 24 h were retrospectively screened to identify cases of RSE or SRSE with documented and analyzable electrographic seizures. RSE was defined as SE persisting despite adequate doses of benzodiazepines followed by an appropriate ASM (Au et al. 2024).

EEG reports were reviewed, and patients were classified into two groups: those with cyclic seizures, defined by a regular recurrence interval (cyclic seizures patients [CSp]), and those with seizures without an identifiable cyclic pattern (noncyclic seizures patients [NCSp]), who served as the control group. The control group included patients with RSE or SRSE, exhibiting electrographic seizures without a regular recurrence pattern, admitted during the same study period.

Patients without any electroclinical and/or electrographic seizure relapses within the first 48 h after admission, with artifacted EEG recordings or seizures/SE in a postanoxic context, were excluded.

### 2.2 Clinical Variables

Medical records were reviewed for demographics, preexisting neurological conditions, brain injury mechanism, SE severity, and initial management. The Charlson comorbidity index was used to assess comorbidities (Charlson et al. 1987). SE etiologies were categorized as acute, remote, progressive brain injury, and encephalopathy (toxic, metabolic) (Trinka et al. 2015). Clinical and biological variables were collected upon ICU admission, including previous and ongoing treatments during cEEG monitoring, as well as medications interfering with seizure threshold (referred to as interfering treatments). The Simplified Severity Index II Score (IGS II) was calculated within the first 24 h. ICU stay and mechanical ventilation duration were recorded. Mortality and functional outcome (GOS and mRS scores) at discharge and at 6‐month later were obtained. Poor outcomes were defined as a GOS of 1–3 and/or an mRS score of 3–6.

### 2.3 EEG Acquisition, Monitoring, and Analysis

**FIGURE 1:**
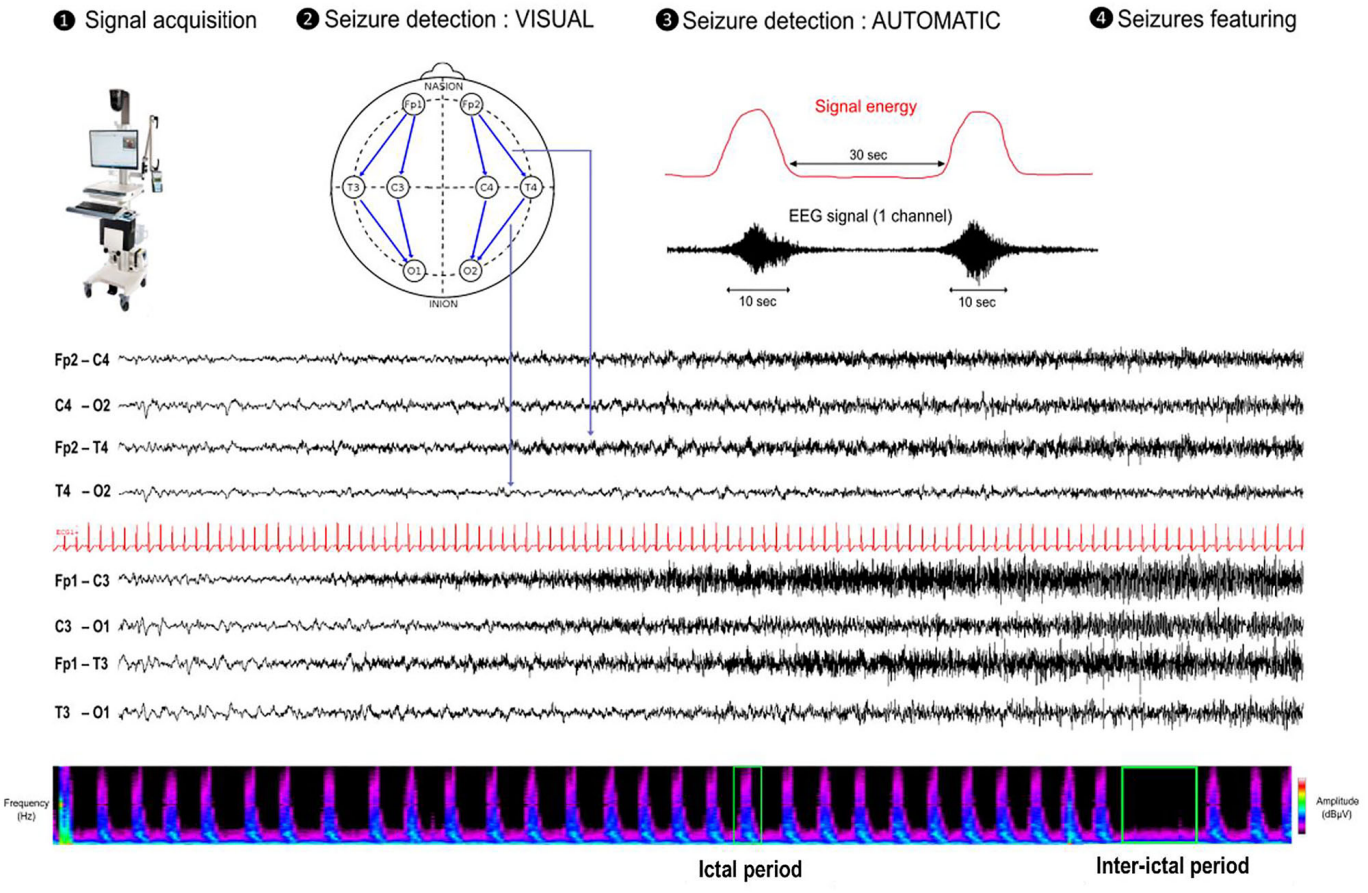
**EEG acquisition, automatic seizure detection, and feature extraction**. Upper panel: Electrode placement and montage used eight recording electrodes (four paired electrodes *(frontal FP1–FP2; central C3–C4; temporal T3–T4; and occipital O1–O2), following a modified 10–20 system*. Reference and ground electrodes were placed on the median line of the scalp. An electrocardiogram electrode was additionally placed for single‐lead EKG. The main steps of the automatic seizure detection procedure were as follows: (1) EEG segmentation into 10‐s windows, with 5‐s overlap. (2) Bandpass filtering between 1 and 40 Hz reduces line noise and muscle artifacts. (3) Detection performed on single EEG channels, with epileptic discharges identified by thresholding the total energy level across all channels. (4) Application of the detection module to all overlapping 10‐s segments. Lower panel: Raw EEG of a seizure, showing focal onset in the left anterior region, characterized by fast rhythms, propagating to the right hemisphere *(sampling recording rate: 256 Hz, low‐pass filter: 70 Hz, high‐pass filter: 0.53 Hz, notch filter: 50 Hz, sensitivity: 100 µV/cm)* and a 3‐hour time‐frequency chart (left: right hemisphere, right: left hemisphere; *voltage spectrum 0–40 Hz*) based on compressed spectral array density showing several repetitive seizures (i.e., a cluster of cyclic seizures). EEG, electroencephalogram monitoring.

Continuous video‐EEGs were recorded using a standard clinical digital video‐EEG system (Micromed, System PLUS Evolution 1.04.95), with 256 Hz sampling and 8 silver chloride electrodes according to the 10–20 system (Figure 1).

#### 2.3.1 Seizures Detection

Seizures were defined according to ACNS standardized critical care EEG terminology (Hirsch et al. 2021), requiring ictal discharge lasting at least 10 s and be separated by at least 30 s. Clinical correlates were evaluated through video review or EEG interpretation charts. The EaSiBUSSEs score was calculated to assess the severity of SE and the risk of seizure recurrence (Hanin et al. 2021).

##### 2.3.1.1 Visual (Raw EEG) and Quantitative (CSA) Seizure Detection Methods

Color spectral array (CSA) was used to detect seizures or amplitude changes in preictal, ictal, and postictal states. All recordings were reviewed by both a clinician and a neurophysiologist for inter‐rater agreement on ictal onset. Visual inspection of raw EEG data was the gold standard for detection.

##### 2.3.1.2 Automatic Seizure Detection Method

In addition to manual EEG annotations, seizure detection on multichannel EEGs was performed, based on instantaneous energy measures derived from the Hilbert transform envelope (Esteller et al. 2005). The algorithms were implemented in MATLAB (Mathworks, Natick, MA).

#### 2.3.2 Seizures Classification

**FIGURE 2:**
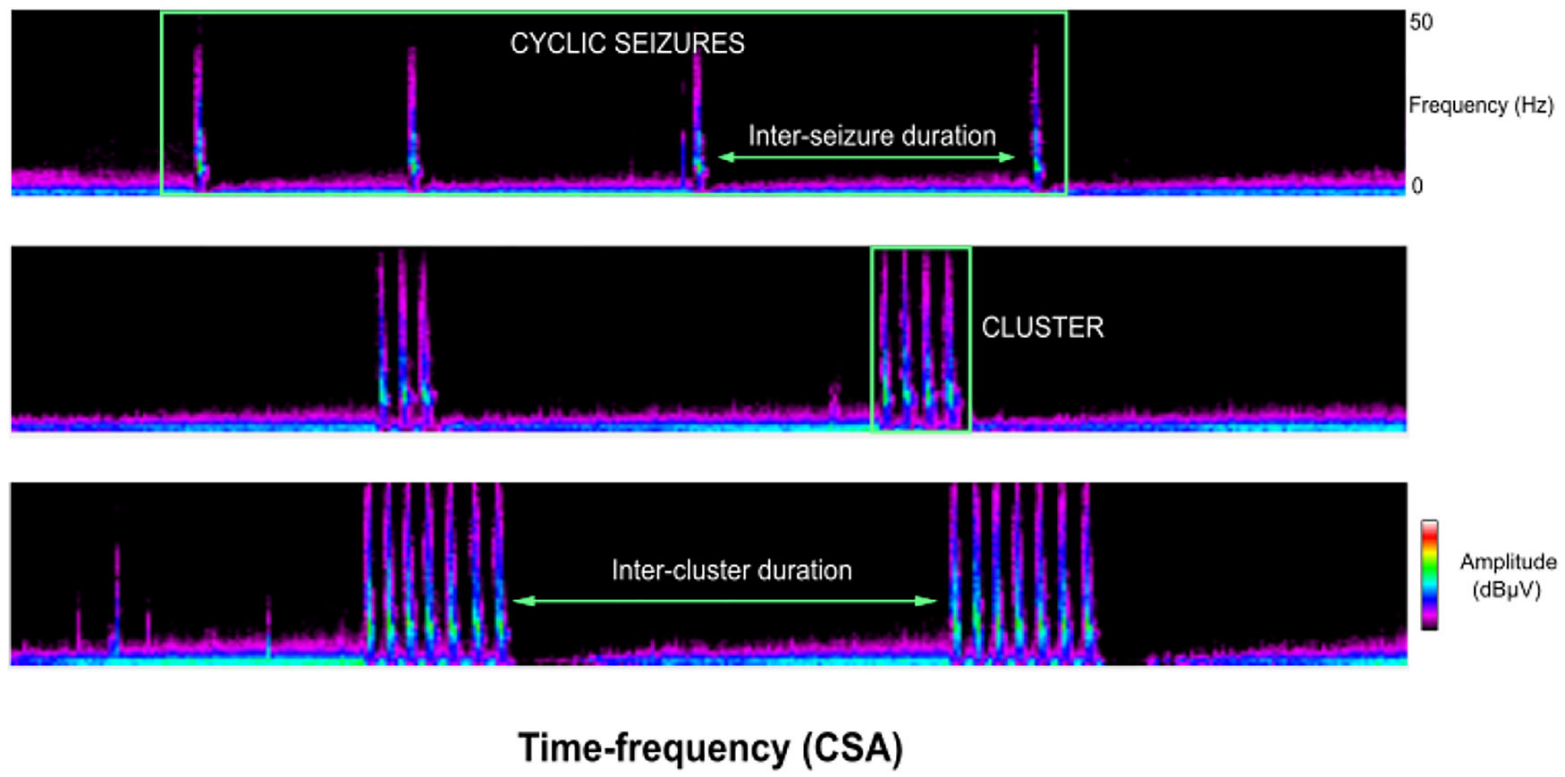
**Representative example of cyclic seizures on a 3‐h compressed spectral array (CSA) chart**. Time‐frequency analysis shows successive seizures occurring in clusters, gradually increasing over time before stabilizing for extended periods. Spectral power analysis indicated that cyclic seizures predominantly consist of high‐frequency activities (11.9 ± 5 in CSp vs. 4.5 ± 3 Hz in NCSp). CSA analysis demonstrates a characteristic EEG interictal pattern: postictal amplitude depression is followed by a peak at the ictal phase, followed by baseline attenuation, which gradually rises again prior to the next seizure. Axes and scale: *Y*‐axis: frequency, *0 Hz at bottom, 40 Hz at the top*. *X*‐axis: 3‐h period. Color scale: power of given frequency, *measured in µV^2^/Hz*.

Cyclic seizures were defined as recurrent seizures occurring at regular intervals, with a minimum of three seizures per hour for at least one hour (Pinto et al. 2017). Cyclic seizures were further subdivided into two categories: (i) discrete (or single) episodes, defined as isolated cyclic seizure events, repeating within a single series, separated by interictal intervals (Figure 1), and (ii) in‐cluster seizures, defined as groups of seizures occurring in close succession within a compressed time frame, also separated by interictal intervals, and potentially recurring multiple times during the recording period. In some cases, these clusters exhibited a double periodicity, with recurrence patterns observed both at the level of individual seizures and at the level of the clusters themselves (Figure 2). Patient profiles were defined by the presence or absence of cyclic seizures, and the proportion of cyclic seizures among all seizures was noted for each patient.

Cyclicity was assessed visually using quantitative EEG trends and confirmed by calculating the inter‐seizure intervals (ISIs). Regularity of seizure recurrence was assessed using the variance of the logarithmic ratio between two consecutive ISIs, according to the following criterion:

$$\mathrm{Var}\;\left[\log\;\left(\mathrm{IS}I_{i}/\mathrm{IS}I_{i-1}\right)\right]<0.5\;\mathrm{and}\;\mathrm{IS}I_{i}<1200\;s$$

where ISI*_i_* is the inter‐seizure interval between seizure *i* and *i* + 1 with a minimum of three seizures per hour required to compute this measure. ISI values correspond to the duration (in seconds) between the onsets of two consecutive seizures.

The distinction between discrete episodes and in‐cluster seizures was made when the inter‐seizure duration was less than two‐thirds of the preceding interval.

#### 2.3.3 EEG Features

Seizure duration was determined through visual examination of raw EEGs. Seizure burden was supported by the total number of seizures indexed to the neuro‐ICU length of stay (seizure daily index), the median time in ictal state (cumulative hours containing at least one seizure relative to total cEEG monitoring time), seizure frequency (seizures per hour), and the occurrence of seizures reported prior to cEEG monitoring.

Ictal epochs were characterized by seizure duration, onset location, ictal zone propagation, peak frequency in the power spectrum, and instantaneous energy. Seizure clusters were detailed by the number of clusters, number of seizures per cluster, duration, and cyclicity (inter‐seizure and inter‐cluster durations). Interictal epochs were characterized by inter‐seizure duration, instantaneous energy before ictal onset (during a 10‐s segment) and after seizure termination (during a 15‐s segment), and severity using the EaSiBUSSEs score (Hanin et al. 2021). Specific interictal EEG patterns were described, including burst‐suppression patterns, periodic activities, epileptiform discharges, or ictal‐interictal continuum patterns. Intra‐patient variability was assessed using a coefficient of variation for seizure and inter‐seizure durations.

### 2.4 Statistical Analysis

All statistical analyses were performed in R (version 4.3.2; R Core Team, 2023). Comparisons between CSp and NCSp groups were conducted across demographic, seizure‐related, severity, and outcome variables. Fisher's exact test was used for categorical variables, and the Wilcoxon rank‐sum test for continuous variables. EEG features were compared across seizure classifications using linear mixed‐effects models (LMMs). Seizure category was included as a fixed effect, and patient identifier was modeled as a random intercept to account for within‐subject pairing across categories. All models were fitted using the lme4 package (v1.1‐35.1), and significance was assessed with Type II Wald chi‐square tests from the car package (v3.1‐2). Post hoc pairwise comparisons were performed with the emmeans package (v1.8.9) using Tukey adjustment for multiple comparisons. Model assumptions, including residual normality and homoscedasticity, were checked using the ggResidpanel package (v0.3.0), and model complexity was evaluated to ensure that the fixed and random effects were supported by the data. All tests were two‐sided, and statistical significance was set at p or adjusted *p* < 0.05.

## 3 Results

Of the 138 patients admitted to the neuro‐ICU for SE over 5 years and monitored for at least 24 h, 16 (12%) exhibited cyclic seizures (CSp), occurring as discrete episodes and/or clusters. They were compared with 15 controls with exclusively sporadic seizures (NCSp). Patients without any electrographic and/or electroclinical seizure relapses after admission were excluded.

### 3.1 Demographics and Clinical Variables

Demographic and clinical characteristics are summarized in Table 1.

**TABLE 1:** Main demographic and clinical characteristics of status epilepticus (SE) patients with cyclic seizures (CSp) and without cyclic seizures (NCSp).

	Patients with cyclic seizures (CSp)	Patients with noncyclic seizures (NCSp)	
	*n = 16*	*n = 15*	*p* value
**Demographics**			
Age, years—*mean (sd)*	31 (15.4)	52 (20)	**0.002**
Sex, male—*n* (%)	12 (75)	6 (40)	0.073
Neurological or Neurosurgical History, yes*—n* (%)	7 (43.8)	9 (60)	0.479
Previous epilepsy, yes—*n* (%)	4 (25)	8 (53.3)	0.28
Psychiatric disorders, yes—*n (%)*	4 (25)	6 (40)	0.458
History of substance abuse, yes—*n (%)*	3 (18.8)	3 (20)	1.000
Charlson index—*mean (sd)*	0.8 (2)	3.5 (2.5)	**<0.001**
Number of interfering treatments^a^—*mean (sd)*	2.2 (1.8)	0.9 (0.9)	**0.032**
**Clinical variables and severity**			
GCS score at admission—*mean (sd)*	10.9 (4.8)	11.6 (2.8)	0.674
FOUR score at admission—*mean (sd)*	10 (5.5)	9 (4.5)	0.67
IGS2 score—*mean (sd)*	31.8 (11.3)	32.9 (12.2)	0.69
ICU length of stay, days—*mean (sd)*	88.2 (76.8)	45.3 (39.7)	**0.04**
Neuro‐ICU length of stay, days—*mean (sd)*	64.6 (49.7)	40.8 (39.4)	0.138
**Status epilepticus (SE)**			
Identified triggering factor, yes—*n (%)*	11 (68.8)	7 (46.7)	0.285
Place of SE definitive diagnosis—*n (%)*			0.564
‐ Out of hospital	3 (18.8)	2 (13.3)	—
‐ Emergency department	2 (12.5)	0	—
‐ Hospital ward	11 (68.8)	13 (86.7)	—
Clinical correlates of SE—*n (%)*			0.28
‐ Generalized	7 (43.7)	7 (46.7)	—
*If generalized, bilateral tonic‐clonic/absence seizure*	*7 (43.7)/0*	*7 (46.7)/0*	*—*
‐ Focal	14 (87.5.)	13 (86.7)	—
*If focal, with impaired consciousness seizure*	*6 (37.5)*	*1 (6.7)*	*—*
‐ Both	5 (31.2)	5 (33.3)	—
Initial therapeutic management of SE—*n (%)*			0.514
First‐line ASMs	12 (85)	13 (87)	—
‐ Second‐line ASMs	14 (87.5)	10 (76.9)	—
‐ Third‐line ASMs	16 (100)	15 (100)	—
Mechanical ventilation duration, days—*mean (sd)*	72.9 (77.3)	30.1 (19.4)	**0.042**
Etiology—*n (%)*			**0.046**
‐ Acute symptomatic^b^	11 (68.8)	3 (20.0)	—
‐ Remote symptomatic^c^	1 (6.2)	7 (46.7)	—
‐Progressive symptomatic^d^	1 (6.2)	2 (13.3)	—
‐ SE in defined electroclinical syndromes	3 (18.7)	3 (20.0)	—
‐ Unknown/cryptogenic	0	0	—
NORSE, yes—*n (%)*	9 (56.2)	3 (20)	0.066
SE grade of severity—*n (%)*			**<0.001**
‐ Refractory	0	6 (40)	—
‐ Super‐refractory	0	4 (26.7)	—
‐Prolonged super‐refractory (>7days)	16 (100)	5 (33.3)	—
SE duration, days—*mean (sd)*	63.2 (62.7)	27.5 (18.1)	**0.05**

Abbreviation: ASMs, antiseizure medications. Statistically significant values are shown in bold.

^a^ Interfering treatments included certain antibiotics (e.g., beta‐lactams and fluoroquinolones), antivirals (e.g., acyclovir), antipsychotics including neuroleptics, specific antidepressants, lithium, opioids including tramadol, beta‐blockers, corticosteroids, baclofen, as well as various immunosuppressive and antineoplastic agents.

^b^ Acute SE etiologies: In the CSp group, these comprised eight cases of autoimmune encephalitis, one PRES, one infectious encephalitis, and one severe hypoglycemia. In the NCSp group, one patient had infectious encephalitis, and two had metabolic causes.

^c^ Remote SE etiologies: In the CSp group, one case followed post‐encephalitis. In the NCSp group, remote causes included one neurosarcoidosis, one mitochondrial disease (sequelae), one post‐encephalitis, and four vascular lesions.

^d^ Progressive SE etiologies: In the CSp group, one case was related to a chronic inflammatory lesion. In the NCSp group, progressive causes included one progressive brain tumor and one neurodegenerative dementia.

Most patients in both groups were transferred from external ICUs for RSE/SRSE monitoring. CSp patients were younger (31 vs. 52 years, *p* = 0.002) and more frequently male (75% vs. 40%, *p* = 0.073). Preexisting neurological conditions were comparable between groups, but CSp had significantly fewer comorbidities (Charlson index 0.8 vs. 3.5, *p* < 0.001).

Initial seizure semiology was similar: approximately one‐third experienced both focal and generalized seizures at onset, with focal motor seizures being more common (*p* = 0.24). Other initial SE presentations included focal non‐motor seizures, often associated with behavioral changes or impaired vigilance, confirmed by EEG findings in some cases. The delay between symptom onset and clinical suspicion of SE was identical (17 days, *p* = 0.15).

Before ICU admission, most patients were under observation in a medical ward, with prior intermittent video‐EEGs documenting an increasing electroclinical or electrographic seizures (17.9 vs. 11.9 seizures, *p* = 0.21).

Acute SE etiologies were significantly more frequent in the CSp group (68.8 vs. 6.7, *p* = 0.001), mainly due to autoimmune encephalitis (including anti‐NMDA receptor, anti‐GAD, and seronegative cases) and NORSE. None of the NCSp patients had autoimmune encephalitis. Consequently, immunotherapy was administered far more frequently in CSp (75% vs. 6.7%; *p *< 0.001). Remote SE etiologies were more common in the NCSp group. No SE case in our cohort was classified as having an unknown etiology.

### 3.2 SE Treatments

Midazolam was the first hypnotic agent used in all patients. Although early SE management did not significantly differ between groups, CSp more often received ketamine (68.8% vs. 33.3%) and thiopental (31% vs. 20%). CSp were exposed to a greater number of anesthetic agents (2.8 vs. 2.1 CIVADs) and ASMs (5.8 vs. 5.6). Half of the CSp required three or more CIVADs, typically combinations of midazolam, propofol, ketamine, and/or thiopental; one‐third of CSp did not receive propofol. In NCSp group, most received two CIVADs (53%), and none required more than three. Ketogenic diet was initiated in 31% of CSp versus 6.7% of NCSp. No inhaled anesthetics were used.

Cyclic seizures occurred exclusively in SRSE or prolonged SRSE, and CSp had longer durations of general anesthesia (63 vs. 27 days, *p* = 0.05) and mechanical ventilation (72.9 vs. 30.1 days, *p* = 0.042) and ICU stay (88.2 vs. 45.3 days, *p* = 0.04). Before neuro‐ICU admission, CSp had been exposed to more pro‐convulsive treatments (*p* = 0.032).

### 3.3 EEG Features

A summary of seizure burden and EEG features is provided in Table 2.

**TABLE 2:** Main EEG features of status epilepticus (SE) patients with cyclic seizures (CSp) and SE patients without cyclic seizures (NCSp).

	Patients with cyclic seizures (CSp)	Patients with noncyclic seizures (NCSp)	
	*n = 16*	*n = 15*	*p* value
**cEEG monitoring**			
Duration, days—*mean (sd)*	35.8 (36.9)	9 (4.4)	**0.003**
**Seizures burden**			
Number of seizures prior to cEEG	17.9 (16.9)	11.9 (6.6)	0.214
Total number of seizures	454 (466)	20.2 (25)	**0.002**
Number of seizures according to LOS (index)	12.3 (15)	1.2 (2)	**0.01**
EaSiBUSSEs score	6.9 (0.3)	5.9 (0.9)	**<0.001**
STESS score	3.2 (0.9)	3.1 (0.9)	0.54
**EEG features**			
Classification of seizures according to their type			—
Cyclic/In‐cluster/Sporadic—*number (%)*	212.4 (44)/196.3 (40)/78.9 (16)	0/0/20.2 (100)	—
Clinical correlates of seizures			0.89
‐ Yes	15.5	20.8	—
‐ No	84.5	79.2	—
‐ Both	34.6	25	—
Frequency of seizures			**0.011**
‐ Slow	31.9	73.6	—
‐ Rapid	54.5	15.6	—
‐ Both	13.6	10.8	—
Duration of seizures (s)—*mean (sd)*	68.8 (52.4)	57.9 (41.9)	0.56
Ictal onset location *(%)*			0.172
Diffuse	8.5	6.7	—
‐ Focal	76.7	86.6	—
‐ Both	14.8	6.7	—
if focal, preferential location *(%)*			0.317
‐ Anterior	44.5	23.5	—
‐ Posterior	11.1	5.9	—
‐ Central	5.5	5.9	—
‐ Multiple locations	38.9	64.7	—
if focal, preferential side *(%)*			0.369
‐ Right	33.4	57.4	—
‐ Left	46.6	35.8	—
‐ Both	20	6.8	—
if focal, bilateralization *(%)*			**0.006**
‐ Yes	61.5	7.2	—
‐ No	7.8	50	—
‐ Both	30.7	42.8	—

Abbreviations: cEEG, continuous EEG; LOS, length of stay; s, seconds; sd, standard deviation. Statistically significant values are shown in bold.

#### 3.3.1 Seizure and Interictal Burden

cEEG monitoring was significantly longer in CSp (35.8 ± 36.9 vs. 9 ± 4.4 days, *p* = 0.003). They experienced markedly more seizures (mean 454 vs. 20.2; *p* = 0.002), with maximum of 1636 versus 100, and a higher daily seizure index (12.3 vs. 1.2, *p* = 0.01). Median cumulative ictal time reached 36% of the recording period (range 5%–87.5%).

Interictal EEG patterns and global seizure burden, assessed by EaSiBUSSEs score, were significantly higher in CSp (6.9 out of 7 vs. 5.9, *p *< 0.001), indicating increased seizure risk in the following 24 h for CSp. All patients displayed interictal epileptiform discharges, 78% showed periodic activities, and over half exhibited a burst‐suppression pattern (69% vs. 31%). Ictal‐interictal continuum patterns were more prevalent in CSp (75% vs. 25%).

#### 3.3.2 Cyclic Seizures Characteristics

All CSp had discrete cyclic seizures, accounting for 44% of all events. All but one also displayed in‐cluster seizures (40%), though clustering patterns varied among individuals. The remaining 16% were noncyclic (sporadic).

The majority of seizures were electrographic non‐motor seizures, occurring in 84.5% of CSp and 79.2% of NCSp. Motor manifestations (tonic ± myoclonic seizure) were the most common clinical sign when seizures were clinically apparent. Ictal EEG discharges were typically focal with anterior onset in both groups (82%). Spectral power analysis indicated that cyclic seizures predominantly consisted of high‐frequency activity (11.9 ± 5 Hz in CSp vs. 4.5 ± 3 Hz in NCSp).

There was no significant difference in duration between cyclic and noncyclic seizures (69 vs. 58 s, *p* = 0.56), with interictal intervals averaging 6 min (range 3–15).

Within individual patients, seizure durations and recurrence intervals were remarkably consistent, whereas inter‐patient cyclicity was highly variable, suggesting individualized temporal patterns. In some cases, seizures or clusters occurred at varying periodicities during their ICU stay.

Cyclic seizures were more likely to propagate to the contralateral hemisphere (61.5% vs. 50%, *p* = 0.006).

#### 3.3.3 Clusters

**FIGURE 3:**
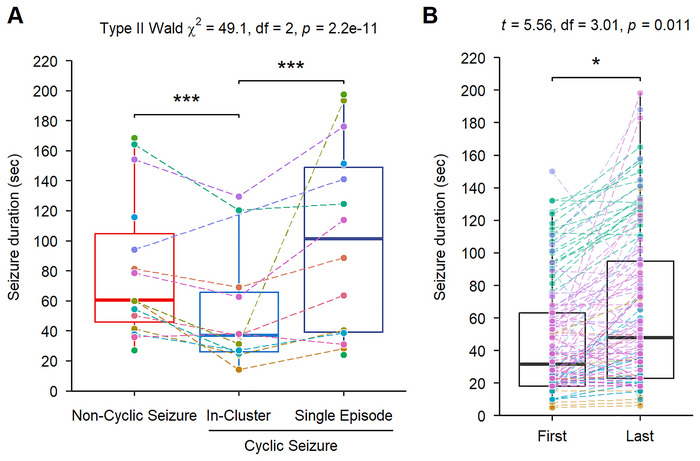
**Comparison of seizure durations in patients with and without cyclic seizures**. (A) Comparison of seizure durations across different seizure types: noncyclic, cyclic, and cluster seizures. (B) Comparison of seizure durations between the first and the last seizure within a cluster, illustrating intra‐cluster evolution.

Clusters represented over one‐third of all cyclic seizures, reaching up to 88% in some patients. The mean number of clusters was 51.7, with a maximum of 326. Each cluster contained an average of 3.2 seizures, with up to 91 in a single cluster. Seizures within clusters were shorter than discrete cyclic or noncyclic seizures (100 and 70 s, respectively, *p* < 0.001). The first seizure in a cluster was shorter than the last (32.1 vs. 48.7 s, *p* = 0.01, Figure 3).

The mean inter‐seizure duration within a cluster was 2.15 min, compared to 15.7 min before cluster onset. Most clusters occurred periodically with a mean inter‐cluster interval of 15.5 min.

### 3.4 Outcome of SE Patients According to the Occurrence of Cyclic Seizures

Mortality rates at ICU discharge (18.7% vs. 20%) and at 6 months (33.3% vs. 31.2%, *p* = 1) were similar in both groups.

Baseline functional status differed between groups, with NCSp showing slightly worse functional status at SE onset (mRS at *T*_0_: CSp 0.7 vs. NCSp 1.9, *p* = 0.004; GOS at *T*_0_: CSp 4.7 vs. NCSp 4.1, *p* = 0.02).

**FIGURE 4:**
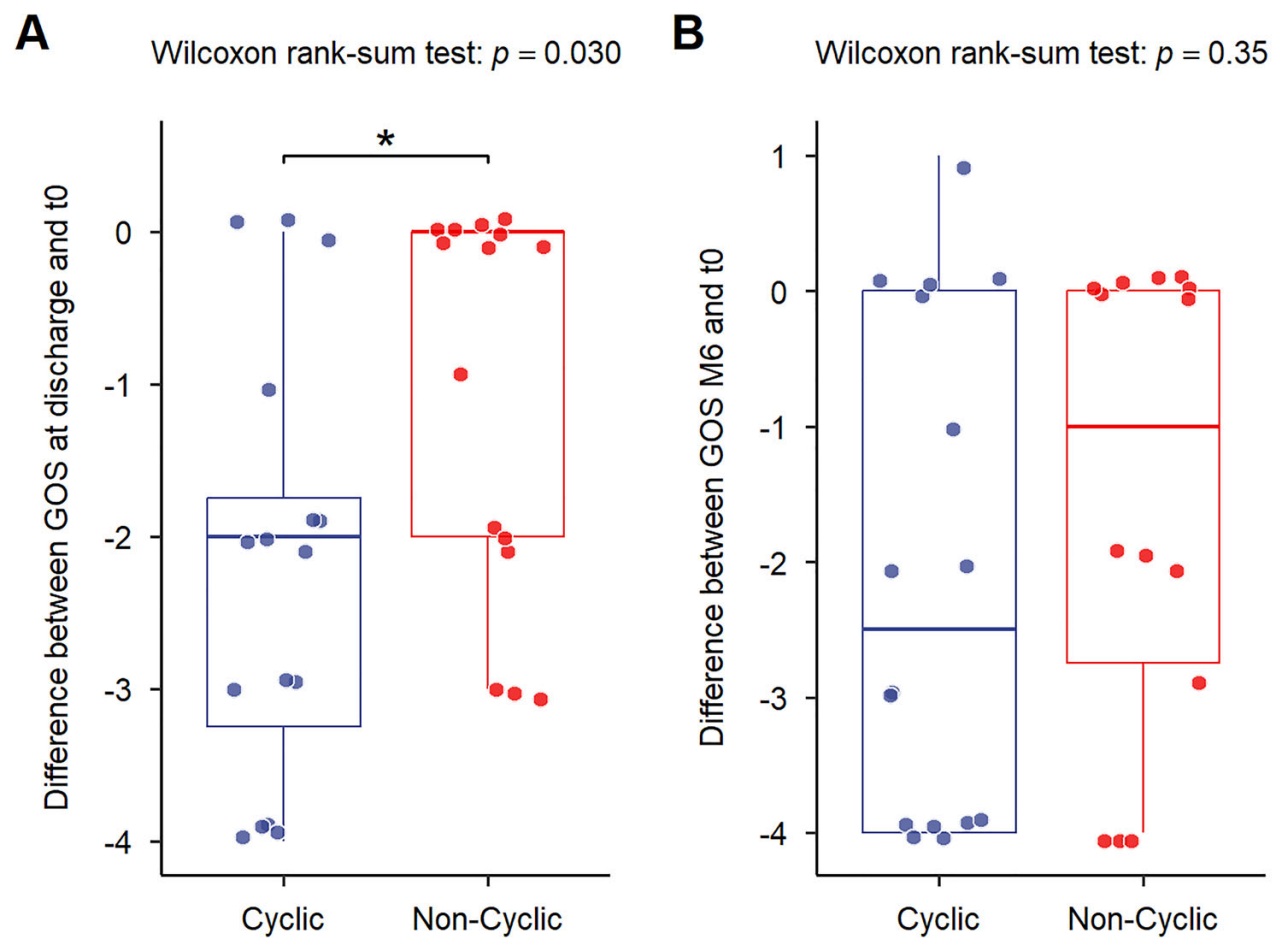
**Comparison of functional outcomes in patients with and without cyclic seizures**. (A) Changes in functional outcome (∆GOS), from admission (*t*_0_) to discharge, comparing CSp and NCSp. (B) Changes in functional outcome based (∆GOS), from admission (*t*_0_) to 6‐month follow‐up, comparing CSp and NCSp.

Among survivors, CSp showed a greater functional decline at discharge (∆GOS scores: −2.2 vs. −1.1 in NCSp, *p* = 0.02), though this difference was no longer significant at 6 months (*p* = 0.34) (Figure 4). Mean GOS and mRS scores indicated poorer shorter recovery in CSp (discharge: 2.4 vs. 4; 6 months: 2.6 vs. 3.75). Moreover, CSp developed more severe seizure‐related sequelae, as indicated by the increased number of ASMs at discharge (*n* = 4.4 vs. 3.3).

## 4 Discussion

In this study, cyclic seizures were observed in 16 out of 138 patients monitored for suspected SE, over a 5‐year period, representing 12% of cases, consistent with previous reports (Pinto et al. 2017; Zorlu et al. 2021). Extended monitoring (median 35 days) allowed a more comprehensive evaluation of their natural history and temporal dynamics. Notably, more than 80% of CSp exhibited no clinical motor manifestations, highlighting the importance of continuous monitoring to detect electrographic non‐motor seizures (Claassen et al. 2004; Kubota et al. 2018).

These seizures typically occurred early in SE but could recur after a period of controlled epileptic activity, supporting previous findings that 80% of seizures in comatose patients occur within the first 24 h (Kubota et al. 2018; Limotai et al. 2019). Although a minimum of 48 h of monitoring is recommended in comatose patients, extended monitoring may be required if epileptiform patterns persist (Rubinos et al. 2020; Outin et al. 2021). The EEG features of cyclic seizures, including short duration (<1 min), high‐frequency discharges, variable interictal intervals (5–10 min), and focal onset, were consistent with prior studies (Friedman et al. 2008; Pinto et al. 2017; Zorlu et al. 2021).

### 4.1 Patient Characteristics and Clinical Course

In both groups, the long delay between symptom onset and clinical suspicion of SE likely reflects the non‐specific symptoms, such as headache, confusion, or subtle behavioral changes, which often precede overt seizures.

CSp were generally younger and had fewer comorbidities, although some of whom had a history of epilepsy, in line with previous research (Friedman et al. 2008). In contrast, other studies have reported older patients with more acute or progressive brain injuries (Pinto et al. 2017). NCSp generally showed a quicker response to treatment, leading to earlier termination of monitoring, whereas CSp were associated with a more severe clinical course, evidenced by a set of variables including an increased number of therapeutic interventions before neuro‐ICU admission, longer ICU stays, and increased mechanical ventilation time.

Importantly, cyclic seizures had a higher risk of progression to prolonged SRSE (≥7 days under anesthetics), a rare condition in NCSp. The presence of cyclic seizures appears to identify a subgroup of patients with greater refractoriness, who are more likely to require extended intensive care. These findings suggest that cyclic seizure patterns may serve as a marker of SE severity, highlighting a clinically relevant aspect of patient heterogeneity. Overall, this may represent the most severe form of SE, lying at the extreme end of a continuum of uncontrolled, treatment‐resistant seizures.

### 4.2 Outcomes

Survivors in the CSp group had marked functional impairment at discharge and a high risk of developing pharmacoresistant epilepsy. However, the small sample size may account for the absence of statistically significant differences in mortality or functional decline (Kämppi et al. 2024). The lack of excess mortality observed in the CSp group may reflect either the use of more intensive therapeutic interventions in these patients or a seizure pattern that is not necessarily more aggressive in the short term, given that CSp patients may experience seizure‐free intervals, unlike continuous seizure activity that typically ceases only with deep anesthesia.

### 4.3 Treatment Burden and EEG Dynamics

Recent studies pointed out the influence of long‐term treatment on seizure outcomes, with prolonged treatments also linked to changes in seizure periodicity (Busl et al. 2022; Trinka et al. 2023). The poorer outcomes in CSp might therefore result from overtreatment, or the treatment burden itself may have influenced the changes in seizure periodicity. Notably, the duration of CIVADs use was about twice as long in CSp. Cyclical interictal discharges are known to increase during burst‐suppression states induced by anesthetic drugs (Ferron et al. 2009; Kroeger and Amzica 2007). Although our study did not explore in depth the evolution of treatments across different seizure stages, it represents an important limitation and a potential focus for future studies investigating risk factors for cyclic seizures and the impact of treatment on seizure periodicity.

### 4.4 Pathophysiology and Seizure Clustering

SE etiology remains a key determinant of prognosis. Refractoriness in CSp may reflect more severe underlying pathology, as evidenced by the overrepresentation of acute causes, such as NORSE and FIRES (Wickström 2023; Haanpää et al. 2022). Autoimmune encephalitis, more frequent in CSp, may predispose to recurrent seizures through neuroinflammatory mechanisms, altering cortical excitability and the excitatory‐inhibitory balance, thereby promoting recurrent seizures and cyclic seizure patterns.

Seizure clustering, classically described in temporal lobe epilepsy, was also present in acute SE causes, notably in NORSE patients without preexisting epilepsy (Blume and Wiebe 1997; Fugate and Rabinstein 2015; Elmali et al. 2021). The electroclinical features associated with seizure clusters may help elucidate mechanisms of seizure initiation and termination (Ghoshal et al. 2017; Navarro et al. 2007). Some authors have suggested that seizure clusters reflect reduced inhibition and increased excitability in the brain. Our findings revealed a discrepancy between the first and terminal intra‐cluster seizure duration, the latter being longer, possibly activating self‐regulatory inhibitory mechanisms to terminate the cluster (Ferastraoaru et al. 2016). Another hypothesis is that recurrent seizures may lower seizure threshold, creating a vicious cycle of cyclic seizures. Seizure clustering persisted over time, consistent with prior findings that seizures become more severe and clustering more pronounced after SE (Lim et al. 2018). Intractable epilepsy is a primary risk factor for seizure clustering, with pharmacoresistant seizures often occurring in clusters. Such seizures tend to trigger further ones (Haut et al. 2002; Jafarpour et al. 2019; Osorio et al. 2009).

Nevertheless, we cannot rule out the possibility that, at a larger time scale, the periodic recurrence of seizures may follow physiological cycles such as hormonal, menstrual, or sleep‐wake cycles, which were not assessed in this study (Fernández and Loddenkemper 2018; Karoly et al. 2018; Baud et al. 2018; Leguia et al. 2021). Some authors have proposed that subcortical structures (thalamus, hypothalamus, and basal ganglia) may initiate or maintain cyclic seizures, whereas others suggest that cortical spread depression promotes seizure periodicity (Busl et al. 2022). Overall, the consistent cyclical nature of seizures could signal an early signature of pharmacoresistant epilepsy.

### 4.5 Limitations

This study has several limitations. First, EEG was performed using 8–10 electrodes which, while suitable for bedside monitoring in critically ill patients, may hinder the detection of focal seizures due to lower spatial resolution, potentially leading to underestimation of seizures with focal onset or propagation patterns. Second, the sample size is small due to the rarity of cyclic seizures. Third, the control group was not matched for clinical variables—introducing a potential source of bias. Despite these constraints, this study provides one of the few comparative analyses of cyclic seizures and offers valuable insights into their characterization and clinical relevance.

### 4.6 Conclusions

Cyclic seizures were associated with a high seizure burden, as evidenced by several hundred recurrent seizures per patient, contributing to worse functional and cognitive outcomes (Kämppi et al. 2024; de Marchis et al. 2016; Payne et al. 2014). Persistent seizures may promote excitotoxicity, neuronal injury, blood–brain barrier dysfunction, and altered neuronal networks, which may result in disabling cognitive sequelae and drug‐resistant epilepsy (Gorter et al. 2015; Hanin et al. 2020). High‐frequency periodic discharges have been associated with reduced brain oxygenation, in line with MRIs findings of peri‐ictal abnormalities and worse outcome or later epilepsy (Witsch et al. 2017; Bonduelle et al. 2023).

In conclusion, cyclic seizure patterns may identify patients at risk of prolonged SRSE and may serve as a surrogate marker of severity or a potential for secondary brain injury. Early detection of cyclic seizures through cEEG may encourage ICU physicians to consider immediate corrective therapeutic interventions, as these patterns may carry significant prognostic value.

## Author Contributions

**Maeva Le Goïc**: conceptualization, investigation, data curation, funding acquisition, project administration, visualization, writing – original draft, formal analysis. **Mario Chavez**: methodology, software, validation, formal analysis, supervision, writing – review and editing. **Sophie Demeret**: supervision, funding acquisition, writing – review and editing, resources. **Meriem Bouguerra**: project administration, data curation, investigation. **Baptiste Crinière‐Boizet**: visualization, formal analysis, software. **François‐Xavier Lejeune**: formal analysis, software, resources, visualization. **Lucas di Meglio**: investigation, resources, writing – review and editing. **Alexandre Ledos**: investigation, visualization, writing – review and editing, validation. **Nicolas Weiss**: investigation, validation, supervision, writing – review and editing, resources. **Vincent Navarro**: writing – review and editing, validation. **Virginie Lambrecq**: conceptualization, methodology, data curation, investigation, validation, supervision, funding acquisition, visualization, writing – original draft, writing – review and editing.

## Funding

Funding was provided by University of the French Antilles, for a year of research during Maeva Le Goïc's internship in intensive care medicine. This work was supported by the DMU Neurosciences AP‐HP, Paris, France.

## Conflicts of Interest

S.D. reports personal fees from UCB outside the submitted work. V.N. reports personal fees from UCB, Jazz Pharma, and Angelini, outside the submitted work. The rest of the authors declare no conflicts of interest.

## Ethics Statement

The authors confirm adherence to ethical guidelines. This study was reviewed and approved by APHP ethic local commissions (project‐ID reference number: 20240327134527).

## Consent

This study was conducted retrospectively, using anonymized data; the requirement for patient consent was waived. This manuscript does not contain any individual person's data.

## Data Availability

The datasets used during the current study are available from the corresponding author on reasonable request.
